# Self-Sufficient Aflatoxin Decontamination System: MOF-Based Composite Membrane with Peroxidase-Mimic and Controlled H_2_O_2_ Generation

**DOI:** 10.3390/toxins17100516

**Published:** 2025-10-20

**Authors:** Xiaofei Cheng, Wenzhong Zhu, Xueting Zhu, Jinmin Zhang, Jia Yang, Huali Wang, Xiaoqin Mo, Chi Zhang, Lina Wu

**Affiliations:** 1 School of Food Science and Pharmaceutical Engineering, Nanjing Normal University, Nanjing 210023, China; chengxiaofei@mengniu.cn (X.C.); zwzup123@163.com (W.Z.); zhuxueting123a@163.com (X.Z.); sunny1999abc@163.com (J.Z.); 2 Inner Mongolia Mengniu Dairy (Group) Limited by Share Limited, Hohhot 010020, China; 3 Yangzhou Center for Food and Drug Control, Yangzhou 225002, China; ziranyuyj@163.com (J.Y.); moxuyue@sina.com (X.M.); 4 China National Center for Food Safety Risk Assessment, Beijing 100000, China; wanghuali@cfsa.net.cn; 5 Key Laboratory of Biotoxin Analysis & Assessment, State Administration for Market Regulation, Nanjing Institute of Product Quality Inspection, Nanjing 210019, China

**Keywords:** aflatoxin, degradation, metal-organic frameworks, nanozyme, composite membrane

## Abstract

Aflatoxin B1 (AFB1) and its metabolite aflatoxin M1 (AFM1) are stable and carcinogenic mycotoxins that are commonly found in dairy products, posing serious food safety concerns. However, conventional degradation methods face limited degradation efficiency and high energy demand. Here, we develop an innovative polyvinylidene fluoride (PVDF) composite membrane incorporating Fe/Co-based metal-organic frameworks (MOF) (Named Fe/Co-MIL-88B(NH_2_)) and CaO_2_ for targeted aflatoxin removal from milk. This system integrates two synergistic mechanisms: (1) hierarchical porous MOF structures enabling superior aflatoxin adsorption capacity and peroxidase-like catalytic activity, and (2) CaO_2_ acts as a controllable-release H_2_O_2_ donor, supplying a steady flux of reactive oxygen species without the addition of exogenous H_2_O_2_. Moreover, the PVDF membrane with mechanical stability offers uniform immobilization of active components, which prevents the aggregation of nanozymes. As a result, the integrated membrane achieves high degradation efficiency for AFB1 and AFM1, exceeding 95% within 60 min. By eliminating external oxidant addition and minimizing collateral nutrient damage, the technology demonstrates remarkable operational stability (>10 cycles) and milk quality preservation capability. This breakthrough establishes an efficient and reusable detoxification method, providing new opportunities for mycotoxin mitigation in dairy products through spatiotemporal control of reactive oxygen species.

## 1. Introduction

Aflatoxins are a group of carcinogenic secondary metabolites predominantly produced by *Aspergillus flavus* and *Aspergillus parasiticus*, posing severe threats to global food safety. Among them, aflatoxin B1 (AFB1) has been classified as a Group 1 carcinogen by IARC due to its extreme hepatotoxicity and mutagenicity [1,2]. Due to its high chemical stability, AFB1 easily remains in conventional foods such as corn, peanuts, and their products. After being ingested by animals, it is oxidized by P450 into the hydroxyl metabolite aflatoxin M1(AFM1) [3]. This metabolite is subsequently secreted into milk, resulting in contamination of dairy products with reported detection rates exceeding 60% in some regions [4,5]. These toxicological effects of AFB1 and AFM1 raise particular concern due to their presence in widely consumed dairy commodities [5,6]. Thus, the efficient degradation of aflatoxins, especially AFB1and AFM1, is essential for protecting public health and ensuring the safety of agricultural and dairy products.

Traditional methods for aflatoxin degradation primarily involve ozone treatment [7], ultraviolet degradation [8,9], and fermentation degradation using fungi or bacteria [10]. However, these methods typically require a large amount of energy consumption and have low applicability [11,12]. For example, after 60 min of ozone treatment, the content of AFM1 in milk and whey concentrate was reduced to 18.9% and 9.9%, respectively [13]. These limitations put an urgent need for the development of low-energy-consumption and high-efficiency strategies for aflatoxin degradation.

In this context, nanozymes have garnered increasing attention for the application in food safety due to their exceptional catalytic activity, stability, and functional versatility [14,15]. Particularly, nanozymes with peroxidase-like activity have demonstrated extensive utility in biology, chemistry, and medicine owing to their ability to activate H_2_O_2_ and generate reactive oxygen species under mild conditions [16]. Metal organic frameworks (MOFs) represent a novel class of composite materials bridging inorganic chemistry and polymer science, characterized by well-defined porous structures formed through coordination interactions between metal ions/clusters and organic molecules [17,18]. This unique structural design endows MOFs with exceptional properties unattainable in conventional metals or polymers, such as ultra-high surface area, tunable porosity, and outstanding compatibility [19,20,21]. Zhang et al. synthesized a Co-MOF catalyst and investigated its catalytic degradation of tetracycline (TC) using peroxymonosulfate (PMS) activation, achieving a removal rate exceeding 90% within 30 min [22]. Therefore, MOFs, with their multiple unique advantages, serve as an ideal nanozyme platform for degrading mycotoxins, significantly enhancing degradation efficiency [23,24].

Although MOF-based nanozymes with peroxidase-like activity have been widely applied in degrading organic pollutants in wastewater, their application in food toxin degradation remains limited due to the reliance on the addition of exogenous H_2_O_2_ to drive catalytic oxidation_._ Furthermore, excessive residual H_2_O_2_ could impact the food quality because of the potential oxidation of other nutrient substances [15]. Moreover, the inherent nanoparticulate nature of nanozymes makes them easy to aggregate in aqueous solutions, which results in reduced catalytic activity, poor recyclability, and secondary pollution [25]. These limitations underscore the need to develop controlled nanozyme-based methods capable of achieving high adsorption and degradation efficiency toward efficient aflatoxin removal, without relying on the addition of external H_2_O_2_.

To address these challenges, we propose a novel strategy that integrates in situ H_2_O_2_ generation and MOF-based nanozyme with peroxidase-like activity into a composite membrane system for the efficient degradation of aflatoxins. Inspired by previous work on the degradation of mycotoxins using MOF materials and their composites with mycelial membranes (with degradation efficiencies reaching 90~98.6%) [26,27,28,29], we anticipate that the degradation efficiency of the constructed Fe/Co-MIL-88B(NH_2_)/CaO_2_/PVDF composite membrane toward AFB1 and AFM1 will reach approximately 95%. This strategy provides a new method for the degradation and reliable analysis of aflatoxins.

## 2. Results and Discussion

### 2.1. Principles of Aflatoxin Degradation

**Scheme 1:**
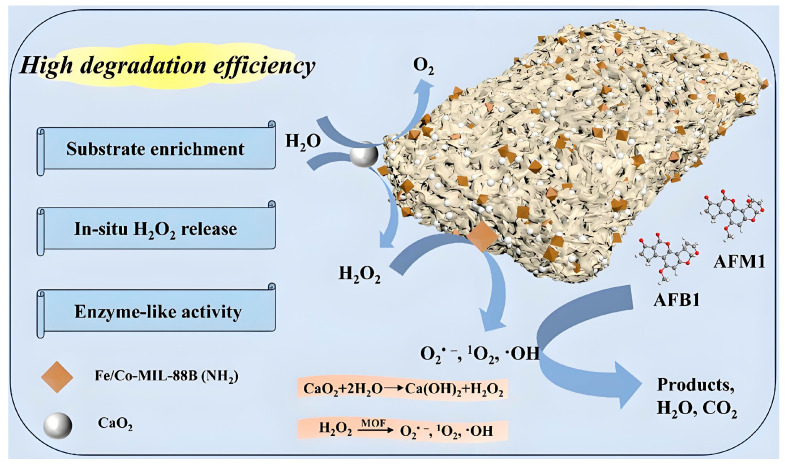
The schematic illustration of the fabricated Fe/Co-MIL-88B(NH_2_)/CaO_2_/PVDF composite membrane for aflatoxin degradation.

We propose a novel strategy for the degradation of aflatoxins that integrates the in situ generation of H_2_O_2_ and MOF-based nanozyme with peroxidase-like activity within a composite membrane system. Specifically, a bimetallic MOF, Fe/Co-MIL-88B(NH_2_), has been synthesized and incorporated into a polyvinylidene fluoride (PVDF) membrane together with CaO_2_ to fabricate the Fe/Co-MIL-88B(NH_2_)/CaO_2_/PVDF composite membrane (Scheme 1). In this integrated system, CaO_2_ enables controlled in situ release of H_2_O_2_, which eliminates the reliance on external addition of H_2_O_2_ and inevitable oxidative damage. Moreover, Fe/Co-MIL-88B(NH_2_) with peroxidase-like activity facilitates the catalysis of H_2_O_2_ to generate reactive oxygen species for aflatoxin degradation. Furthermore, the PVDF membrane not only offers structural integrity but also ensures the uniform immobilization of MOF particles, avoiding the problem of nanoparticle aggregation and poor recyclability. This Fe/Co-MIL-88B(NH_2_)/CaO_2_/PVDF composite membrane achieves a high degradation efficiency for AFB1 (1 mg/L), exceeding 95% within 60 min, outperforming other similar methods in toxin degradation. This work demonstrates an efficient approach for aflatoxin removal, providing valuable prospects for the development of advanced detoxification strategies in the food industry.

### 2.2. Characterization of MOF

**Figure 1:**
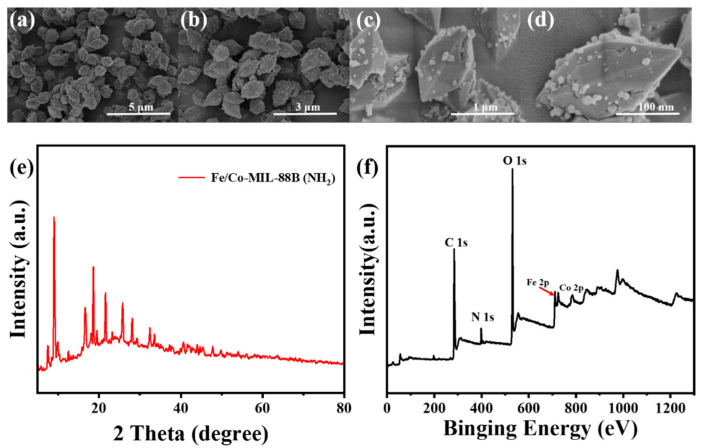
(**a**–**d**) SEM images, (**e**) PXRD pattern, and (**f**) XPS spectra of Fe/Co-MIL-88B(NH_2_). The pattern and spectra are shown for qualitative observation; no quantitative values are implied.

The PXRD pattern of the as-synthesized MOF is consistent with the simulated pattern of MIL-88B, confirming the successful synthesis (Figure 1e). The diffraction peaks observed at 9.2°, 10.1°, 16.5°, and 18.6° represent typical (002), (101), (103), and (200) planes of the MIL-88B structure, respectively [30,31,32,33]. The clear and distinct characteristic peaks associated with each crystalline plane further corroborate the high crystallinity of the synthesized Fe/Co-MIL-88B(NH_2_).

The elemental composition and chemical state of MOF are subsequently investigated by XPS (Figure 1f). The characteristic peaks at 284 eV, 398 eV, and 531 eV belong to C 1s, N 1s, and O 1s, respectively [34]. The Fe 2p spectrum displays peaks at 710.6 eV and 724.3 eV, assigned to Fe 2p_3/2_ and Fe 2p_1/2_, respectively (Figure S1). Additionally, the presence of the satellite peak at 717.1 eV is indicative of Fe^3+^, confirming that Fe exists in the Fe (III) oxidation state within Fe/Co-MIL-88B(NH_2_) [35]. The Co 2p spectrum reveals peaks at 783.3 eV and 795.1 eV (Figure S2), corresponding to Co 2p_3/2_ and Co 2p_1/2_, respectively [36].

The SEM images reveal that Fe/Co-MIL-88B (NH_2_) exhibits a regular octahedral double-cone morphology, with small particle attachments observed on the surface (Figure 1a–d). This morphology closely aligns with the previously reported structure [37], further verifying the successful synthesis of Fe/Co-MIL-88B (NH_2_).

### 2.3. Characterization of Membranes

**Figure 2:**
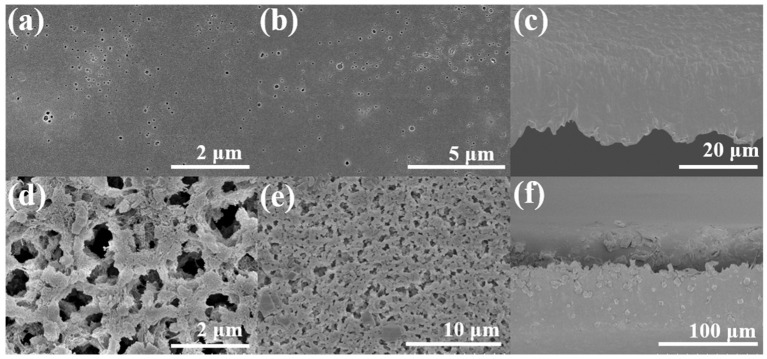
(**a**,**b**) SEM images with the top-down view and (**c**) the SEM image with the cross-section view of the PVDF membrane. (**d**,**e**) SEM images with the top-down view and (**f**) the SEM image with the cross-section view of the Fe/Co-MIL-88B(NH_2_)/CaO_2_/PVDF composite membrane.

Figure 2a–c depicts SEM images of the pristine PVDF membrane, while Figure 2d–f displays those of the Fe/Co-MIL-88B(NH_2_)/CaO_2_/PVDF composite membrane. A significant morphological transformation is observed with the structure evolving from the dense, non-porous architecture of PVDF membrane to the reticulated porous network of composite membrane. The presence of Fe/Co-MIL-88B(NH_2_) particles on the surface of the composite membrane is discernible, indicating successful surface loading. Furthermore, CaO_2_ particles are observed within the membrane cross-section, corroborating the successful preparation of the Fe/Co-MIL-88B(NH_2_)/CaO_2_/PVDF composite membrane.

**Figure 3:**
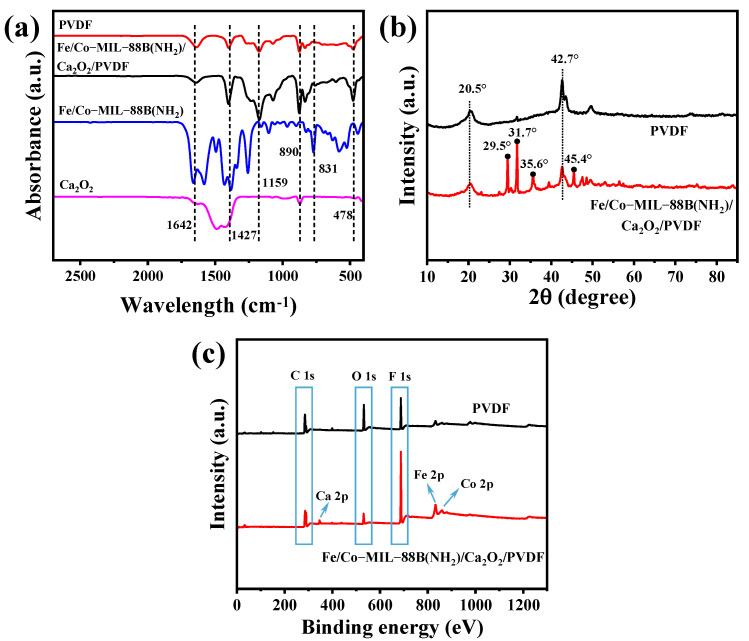
(**a**) The FT-IR spectra, (**b**) PXRD patterns, and (**c**) full-scan XPS spectra of the PVDF membrane and the Fe/Co−MIL−88B(NH_2_)/Ca_2_O_2_/PVDF composite membrane. These spectra are shown for qualitative observation; no quantitative values are implied.

The functional groups present in different membranes were examined using FT-IR spectroscopy. As shown in Figure 3a, the Fe/Co-MIL-88B(NH_2_)/CaO_2_/PVDF composite membrane displays two peaks at 1642 cm^−1^ and 1427 cm^−1^, corresponding to the asymmetric and symmetric vibrations of coordinated carboxyl groups, respectively, indicating successful coordination of the organic ligands with metal centers. The peak at 831 cm^−1^ corresponds to the out-of-plane bending vibration of aromatic C-H in organic ligands, while the low-frequency peak at 478 cm^−1^ is assigned to Fe-O vibrations, confirming the presence of Fe-carboxylate coordination in the MOF structure. These spectral features are consistent with the structural characteristics of MIL-88B [38]. Additionally, the absorption bands 1159 cm^−1^ at and 890 cm^−1^ are attributed to the C-F stretching vibration in the PVDF matrix and the presence of Ca^2+^ binding, respectively [39,40], further confirming the fabrication of composite membrane.

The original PVDF membrane and Fe/Co-MIL-88B(NH_2_)/CaO_2_/PVDF composite membrane are analyzed by PXRD characterization. In Figure 3b, diffraction peaks observed at 20.5° and 42.7° in both membranes correspond to the semicrystalline structure of PVDF [41]. Additionally, the sharp peaks at 29.5°, 31.7°, 35.6°, and 45.4° appear exclusively in the composite membrane, indicating the presence of crystalline phases attributed to the incorporation of Fe/Co-MIL-88B(NH_2_). This phenomenon may be attributed to the shift in diffraction peaks of Fe/Co-MIL-88B(NH_2_) [33].

The elemental composition of the membranes was analyzed by XPS. As illustrated in Figure 3c, compared to the original PVDF membrane, the composite membrane exhibits additional signals related to Fe, Co, and Ca elements, confirming the successful incorporation of Fe/Co-MIL-88B(NH_2_) and CaO_2_.

**Figure 4:**
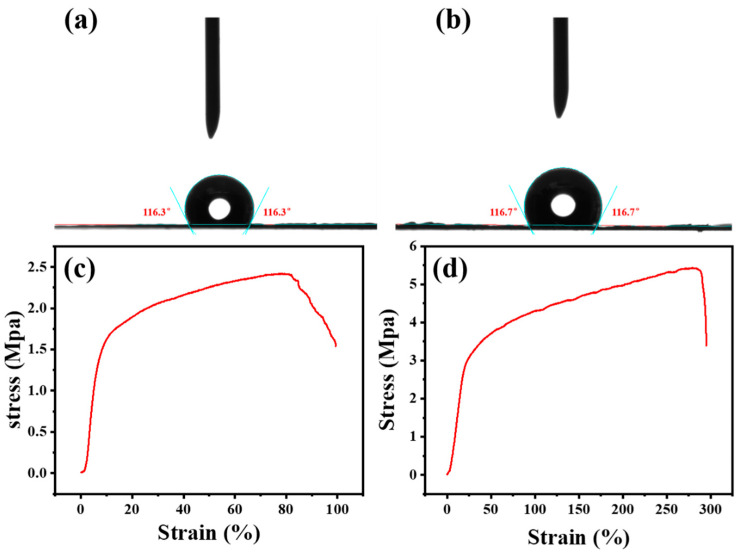
The static contact angles of (**a**) the PVDF membrane and (**b**) the Fe/Co-MIL-88B(NH_2_)/CaO_2_/PVDF composite membrane. The stress–strain measurements of (**c**) the PVDF membrane and (**d**) the Fe/Co-MIL-88B(NH_2_)/CaO_2_/PVDF composite membrane.

To assess the alteration in surface wettability of the composite membrane, static contact angle measurements are conducted on both the original PVDF membrane and the Fe/Co-MIL-88B(NH_2_)/CaO_2_/PVDF composite membrane (Figure 4a,b). The contact angle of the original PVDF membrane is measured at 116.3°, while that of the composite membrane is 116.7°. The minimal changes in water contact angle demonstrate that the incorporation of Fe/Co-MIL-88B(NH_2_) and CaO_2_ has little influence on surface wettability. Moreover, both membranes exhibit contact angles greater than 90°, indicating a hydrophobic characteristic. This inherent hydrophobicity is conducive to maintaining membrane stability in aqueous environments. Additionally, the relatively smooth surface of these membranes also contributes to the high water contact angles as the surface roughness is known to influence wettability [42].

### 2.4. Mechanical Properties of the Membrane

The mechanical properties of the original PVDF membrane and composite membrane are evaluated. As shown in Figure 4c,d and Table S1, the tensile strength, elongation at break, and elastic modulus of the original PVDF membrane are measured to be 2.42 MPa, 99.27%, and 35.5 MPa, respectively. Due to the incorporation of Fe/Co-MIL-88B(NH_2_) and CaO_2_, the composite membrane exhibits significant enhancement in mechanical properties, with elongation at break increasing nearly threefold to 294.98% and the tensile strength doubling to 5.43 MPa. In contrast, the elastic modulus of the composite membrane reduces to 15.87 MPa, implying a reduction in stiffness. These results indicate that the prepared Fe/Co-MIL-88B(NH_2_)/CaO_2_/PVDF composite membrane exhibits significantly improved flexibility, albeit at the expense of reduced rigidity, compared to the original PVDF membrane.

### 2.5. Degradation Condition Experiment

**Figure 5:**
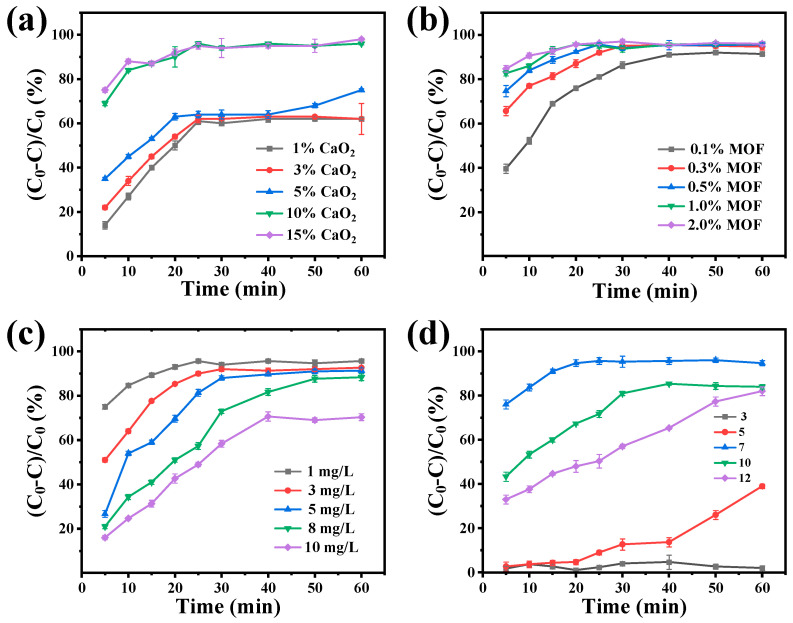
The degradation rate of the Fe/Co-MIL-88B(NH_2_)/CaO_2_/PVDF composite membrane toward AFB1 with different (**a**) CaO_2_ content, (**b**) Fe/Co-MIL-88B(NH_2_) content, (**c**) AFB1 concentration, and (**d**) pH values.

The degradation performance of the Fe/Co-MIL-88B(NH_2_)/CaO_2_/PVDF composite membrane under various conditions is systematically evaluated using a controlled variable method, including changes in CaO_2_ content, Fe/Co-MIL-88B(NH_2_) content, AFB1 concentration, and pH value. As depicted in Figure 5a, the degradation rate of AFB1 gradually increases with increasing CaO_2_ content. However, when the CaO_2_ content exceeds 10% to 15%, the degradation rate reaches a plateau, suggesting that 10% CaO_2_ content may represent a saturation threshold and optimal loading level, given the confines of membrane volume. Figure 5b illustrates the impact of varying Fe/Co-MIL-88B(NH_2_) content in the composite membrane on degradation rate. The degradation rate rises with higher Fe/Co-MIL-88B(NH_2_) content, and it remains consistent at approximately 95% across different MOF contents, though differences become insignificant beyond a content exceeding 0.5%. Therefore, the optimal composition for fabricating composite membranes appears to be 10% CaO_2_ and 0.5% Fe/Co-MIL-88B(NH_2_).

The degradation rate of AFB1 gradually decreases with escalating AFB1 concentration (Figure 5c). Specifically, when the concentration of AFB1 increases from 1 mg/L to 10 mg/L, the degradation rate declines from 95% to approximately 70%. At different pH values, the degradation rate varies (Figure 5d). The composite membrane exhibits the highest degradation rate of AFB1 under neutral conditions, followed by alkaline conditions, and the lowest under acidic conditions. Particularly, at the pH of 3, the composite membrane shows almost no degradation capability toward AFB1. These findings indicate that the composite membrane demonstrates favorable degradability under neutral and mildly alkaline environments. The reduced efficiency under acidic conditions may be attributed to the inhibitory effect of excess H^+^ ions on the enzyme-mimetic activity of Fe/Co-MIL-88B(NH_2_).

To further evaluate the degradation performance of the Fe/Co-MIL-88B(NH_2_)/CaO_2_/PVDF composite membrane, the biodegradation experiment toward AFM1 (1mg/L) is carried out under the optimal degradation conditions of 10% CaO_2_ content, 5% Fe/Co-MIL-88B(NH_2_) content, and a neutral environment, as described in Section 2.5. The results, presented in Figures S3 and S4, demonstrate that the composite membrane achieves a degradation rate of 96% for AFM1 within 1 h, confirming the good degradability of the composite membrane for aflatoxin.

### 2.6. Comparison of Degradation Performance of Different Membrane Compositions

**Figure 6:**
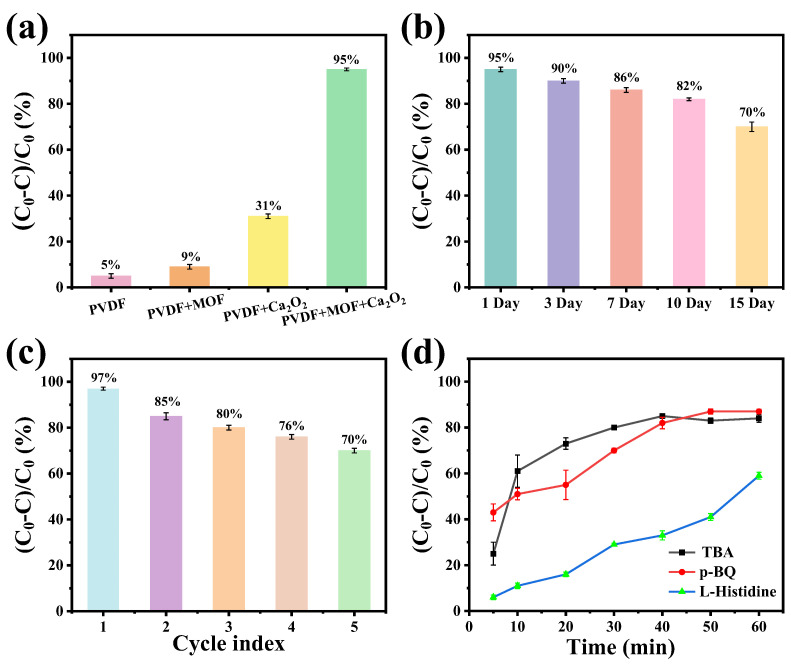
AFB1 degradation changes in different (**a**) membrane compositions (MOF stands for Fe/Co-MIL-88B(NH_2_)), (**b**) Fe/Co-MIL-88B(NH_2_)/CaO_2_/PVDF stability testing, and (**c**) Fe/Co-MIL-88B(NH_2_)/CaO_2_/PVDF cyclable test. (**d**) Degradation rate under different quencher agents.

To investigate the influence of membrane compositions on AFB1 degradation, different material combinations are added during membrane synthesis, including single PVDF, PVDF + Fe/Co-MIL-88B(NH_2_), PVDF + CaO_2_, and PVDF + CaO_2_ + Fe/Co-MIL-88B(NH_2_), respectively (Figure 6a). Neither the PVDF membrane nor the Fe/Co-MIL-88B(NH_2_)/PVDF composite membrane showed any degradation activity toward AFB1. The degradation rate of the PVDF + CaO_2_ composite membrane reaches only approximately 30%, which may be attributed to the oxidative effect of H_2_O_2_ generated from the decomposition of CaO_2_ [11,43]. Notably, with the addition of the Fe/Co-MIL-88B(NH_2_) and CaO_2_, the degradation rate reaches 95%, indicating the synergistic effect between CaO_2_ and Fe/Co-MIL-88B(NH_2_) for effective degradation of AFB1.

### 2.7. Stability and Cyclability of Membranes

The degradation stability of the membrane is crucial for its practical application. Therefore, the stability and recyclability of the membrane are investigated separately. As depicted in Figure 6b, the Fe/Co-MIL-88B(NH_2_)/CaO_2_/PVDF composite membrane exhibits commendable stability during the 15-day test period, with the degradation rate remaining above 75% after 15 days. The decline in degradation rate may be attributed to the reduction in CaO_2_ content within the Fe/Co-MIL-88B(NH_2_)/CaO_2_/PVDF composite membrane during the reaction, leading to a decrease in hydrogen peroxide yield. The degradation rate remains above 80% even after five consecutive cycles, highlighting the excellent recyclability of the membrane (Figure 6c). These results confirm that the Fe/Co-MIL-88B(NH_2_)/CaO_2_/PVDF composite membrane possesses outstanding stability and holds strong potential for long-term practical applications.

### 2.8. Comparison of Metal Leaching Performance

The degradation of pure Fe/Co-MIL-88B(NH_2_) resulted in Fe and Co concentrations of 1272 µg/L and 2533 µg/L, respectively, in the post-reaction solution. In contrast, the degradation of Fe/Co-MIL-88B(NH_2_)/CaO_2_/PVDF composite membrane yielded Fe and Co concentrations below the detection limit, indicating negligible metal leaching. This significantly lower metal concentration is well below the safety limit. It shows that the use of Fe/Co-MIL-88B(NH_2_)/CaO_2_/PVDF composite membrane can effectively prevent the overflow of metal ions and improve safety in food use [44].

### 2.9. Degradation Mechanism

In Figure 6d, the active substances were studied by adding active quenchers to the degradation reaction system. Specifically, *p*-benzoquinone (*p*-BQ), L-histidine, and tert-butyl alcohol (TBA) are added, respectively, each corresponding to O_2_^•−^, ^1^O_2_, and ·OH [45]. It can be found that L-histidine has the greatest impact on the degradation rate, while the other two groups (*p*-BQ, TBA) have relatively minor effects. These results indicate that ^1^O_2_ active substance plays an important role in the degradation reaction, with O_2_^•−^ and ·OH playing secondary roles.

### 2.10. Degradation Products

**Figure 7:**
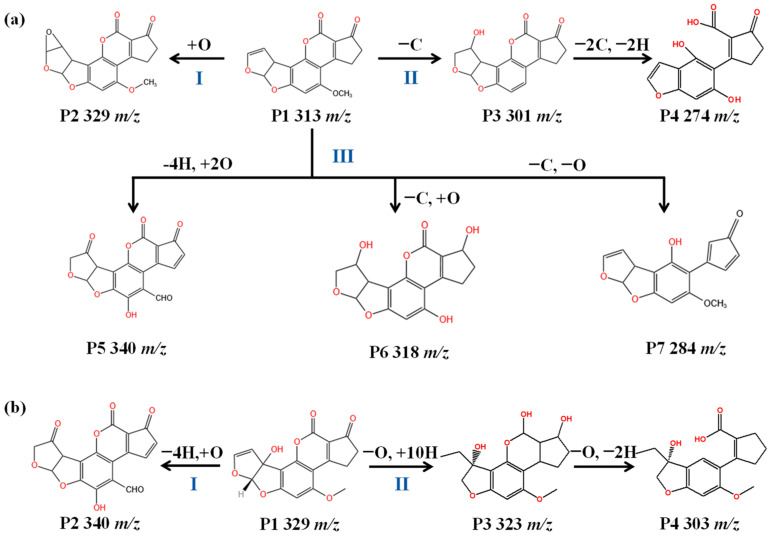
(**a**) AFB1 degradation pathway. (**b**) AFM1 degradation pathway.

The degradation products of AFB1 are analyzed via LC-MS/MS, revealing that the degradation of AFB1 is primarily attributed to alterations in its furan ring and methoxy group. The LC-MS/MS results indicate three distinct degradation pathways. As shown in Figure 7a, Pathway I involves the conversion of P1 AFB_1_ (313 *m*/*z*) to P2 (329 *m*/*z*) through epoxidation of terminal double bonds. Pathway II undergoes hydroxylation and structural modification to generate P3 (*m*/*z* 301). Subsequently, functional group cleavage combined with additional hydroxylation leads to the formation of P4 (*m*/*z* 274) [46]. Pathway III begins with the formation of P5 (*m*/*z* 340) from P1 through oxygen addition to the terminal furan ring and the benzene ring. Subsequent double-bond addition on the furan ring and demethylation of the benzene ring side chain generate P6 (*m*/*z* 318). Finally, the loss of CO results in the formation of P7 (*m*/*z* 284) [47].

The degradation products of AFM1 were further analyzed by LC-MS/MS, revealing two potential pathways (Figure 7b). In pathway I, AFM1 (P1, *m*/*z* 329) undergoes hydration and methoxylation at the terminal furan double bond to generate P2 (*m*/*z* 340) [48]. In pathway II, hydroxylation of P1 introduces two additional hydroxyl groups, leading to the formation of P3 (*m*/*z* 363). This modification likely results from hydration or reductive alteration of double bonds and/or carbonyl groups. P3 is then transformed into P4 (*m*/*z* 303) through dehydration and oxidative rearrangement, accompanied by the loss of hydroxyl groups and the generation of a carbonyl functionality [49].

These metabolites were identified with lower toxicity compared to the parent aflatoxin, suggesting that the degradation process reduces potential health risks. The LC-MS spectra of the degradation products described above are provided in Supporting Information Figures S4 and S5. It should be noted that this work focuses on demonstrating the possible degradation pathways, while the quantitative percentage of each degradation product was not determined.

### 2.11. Safety Test of Composite Membrane

**Figure 8:**
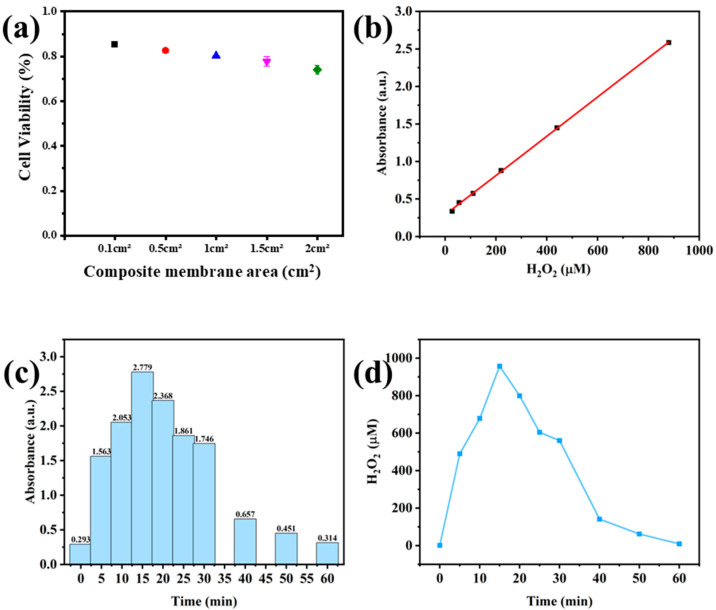
(**a**) HepG2 cell survival after incubation with different membrane areas. (**b**) The linear relationship of H_2_O_2_ concentration and UV-Vis absorption intensity. (**c**) The absorbance of the solution at 352 nm at different times and (**d**) the calculated hydrogen peroxide concentration at different times.

The cytotoxicity of the composite membrane is assessed through cytotoxicological assays, and cell viability is determined via MTT assays. As depicted in Figure 8a, cell survival rates consistently exceed 70% across various areas of the composite membrane. This suggests that the composite membrane exhibits low toxicity and poses no harm to the human body [50]. Examination of the degradation products revealed substantial disruption of the lactone ring, yielding non-toxic small-molecule byproducts [51].

Hydrogen peroxide concentration is monitored over time using UV-Vis spectrophotometry at 352 nm. A pre-established calibration curve (Figure 8b) is employed to convert absorbance readings to H_2_O_2_ concentrations. As depicted in Figure 8c,d, a substantial decrease in hydrogen peroxide levels is observed after 15 min of the reaction. After 60 min, the residual H_2_O_2_ concentration in the solution is approximately 8 µM, significantly lower than the maximum permissible residue limit of 0.5 mg/L for hydrogen peroxide in food products, as established by the U. S. Food and Drug Administration (FDA) [52,53]. Furthermore, the H_2_O_2_ concentration in the reaction solution is significantly lower than the typical 5 mM concentration commonly employed in degradation experiments, which often necessitates exogenous addition [54]. Such findings further affirm the safety of the membrane for use in food applications.

### 2.12. Actual Test of the Composite Membrane

The practical performance of the Fe/Co-MIL-88B(NH_2_)/CaO_2_/PVDF composite membrane is evaluated by introducing AFB1/AFM1 into milk to simulate real-world contamination scenarios. As shown in Figure S6, the degradation efficiencies of AFB1 and AFM1 in milk treated with Fe/Co-MIL-88B(NH_2_)/CaO_2_/PVDF composite membrane exceed 95%, indicating an effective toxin removal. Compared to existing methodologies, including enzymatic degradation and chemical treatments, this composite membrane achieves higher removal efficiency while maintaining milk quality and safety standards (Table S2). This superior performance is attributed to the synergistic catalytic activity of the Fe/Co-MIL-88B(NH_2_)/CaO_2_ component, which facilitates oxidative degradation of aflatoxins under mild conditions. Meanwhile, the initial concentration of the AFB1 and AFM1 used in this study was 1 mg/mL, and experiments confirmed that the constructed composite membrane achieved a degradation rate exceeding 95% for this concentration within 1 h. After treatment by this system, the residual toxin concentration can theoretically be reduced to below 50 μg/mL. The European Commission has set the maximum residue levels (MRLs) for AFB1 and AFM1 in edible foods and milk at 0.5 to 50 ng/mL [55]. The results indicate that this degradation technology can reduce the residual concentration to well below the EU’s maximum residue threshold, sufficiently demonstrating that its efficient toxin removal capability in practical applications meets and significantly exceeds conventional detection requirements. These findings highlight the practical relevance of the membrane for aflatoxin detoxification in milk and provide a basis for further application in food safety.

The results of quality assessments on raw and composite membrane-treated milk samples, conducted using National Food Safety Standard Methods, are summarized in Table S3. The protein, lipid, and lactose content of all three milk brands showed only minor variations following composite membrane treatment. These results further underscore the dependable applicability of the Fe/Co-MIL-88B(NH_2_)/CaO_2_/PVDF composite membrane under practical operating conditions.

## 3. Conclusions

In summary, we have developed a novel ultrathin composite membrane by synergistically integrating Fe/Co-MIL-88B(NH_2_) and CaO_2_ within a PVDF matrix for efficient and safe aflatoxin degradation from milk. This composite membrane simultaneously utilizes the porous structure and peroxidase-like catalytic activity of Fe/Co-MIL-88B(NH_2_), as well as the in situ controlled generation of H_2_O_2_ enabled by CaO_2_, effectively converting aflatoxins in milk into non-toxic small molecules. Moreover, the controlled release of H_2_O_2_ avoids the reliance on additional exogenous H_2_O_2_ and oxidative damage to other nutrients. Furthermore, by incorporating PVDF, the composite membrane eliminates the potential risk of metal ion leaching and nanoparticle aggregation, addressing the concern with the use of MOF-based nanozyme. As a result, the Fe/Co-MIL-88B(NH_2_)/CaO_2_/PVDF composite membrane achieves a superior degradation efficiency of AFB1 and AFM1, exceeding 95% within 60 min, and demonstrates outstanding stability, repeatability, and compactness. This work offers an efficient and reusable strategy for aflatoxin degradation, demonstrating substantial potential for practical application in dairy product processing.

## 4. Experimental Section

### 4.1. Materials

N, N-dimethylformamide (DMF), Ferric (III) chloride hexahydrate (FeCl_3_·6H_2_O), cobalt(II) nitrate hexahydrate (Co(NO_3_)_2_·6H_2_O), 2-Aminoterephthalic acid (H_2_(NH_2_)BDC), AFB1, and AFM1 are obtained from Aladdin Biochemical Technology Co., Ltd. (Shanghai, China). H_2_O_2_ (3%) and CaO_2_ are purchased from Yuanye Biochemical Technology Co., Ltd. (Shanghai, China). PVDF powders (Solef 1010), tert-butyl alcohol (TBA, 99%), *p*-Benzoquinone (*p*-BQ, 99%), L-histidine (99%), Dimethyl sulfoxide (DMSO), and 3-(4,5-dimethylthiazol-2-yl)-2,5-diphenyltetrazolium bromide (MTT) are obtained from Macklin Biochemical Technology Co., Ltd. (Shanghai, China). Potassium iodide (KI) was obtained from Sigma-Aldrich (St. Louis, MO, USA). The whole milk (3.5% fat) is obtained from the Mengniu Dairy. All reagents can be used directly without purification; the aqueous solution is ultra-pure water with a resistivity of 18.2 MΩ.cm.

### 4.2. Preparation of Fe/Co-MIL-88B(NH_2_)

Fe/Co-MIL-88B (NH_2_) is synthesized via a solvothermal method as in previous reports [37]. Specifically, 0.82 g FeCl_3_·6H_2_O, 0.90 g Co(NO_3_)_2_·6H_2_O and 0.53 g H_2_(NH_2_)BDC are dissolved in 31.81 g of DMF. The mixture is stirred for 1 h and then poured into a 100 mL Teflon-lined reactor. The reactor is heated at 120 °C for 24 h in an oven. After the reaction, the powdered material is naturally cooled to room temperature, then washed five times with DMF, centrifuged, and dried at 60 °C for 8 h to obtain Fe/Co-MIL-88B (NH_2_) nanozyme.

### 4.3. Membrane Preparation

Fe/Co-MIL-88B(NH_2_)/CaO_2_/PVDF composite membranes are prepared using the non-solvent induced phase separation (NIPS) method [21] with modification. Initially, powdered Fe/Co-MIL-88B (NH_2_) and CaO_2_ are uniformly dispersed in DMF solvent for 10 min using ultrasonic vibration to prevent significant aggregation of particles in the system. Subsequently, PVDF powder is added to the above solution and dissolved by mechanical stirring at 50 °C for 4 h to form a casting solution. Finally, the casting solution is degassed at a constant temperature for 4 h to eliminate bubbles. The Fe/Co-MIL-88B(NH_2_)/CaO_2_/PVDF composite membrane is then cast onto a glass plate using a membrane coater.

After casting, the membrane is immersed in deionized water and washed several times with deionized water to remove residual solvent. It is then stored in deionized water and replaced with deionized water every 12 h.

### 4.4. Characterization

The morphology and structure of Fe/Co-MIL-88B (NH_2_) and the membrane are observed by scanning electron microscope (SEM, JEOL Ltd, JSM-5610LV, Japan, procuring Country: Nanjing, China). The Fourier-transform infrared (FT-IR) spectrum of Fe/Co-MIL-88B (NH_2_) was recorded using an FT-IR spectrometer (Bruker Corporation, Vertex 70, Germany, procuring Country: Nanjing, China). The spectrum of the composite membrane was recorded using an attenuated total reflection FT-IR (ATR-FTIR, Thermo Nicolet Corporation, Nexus 670, USA, procuring Country: Nanjing, China). The powder X-ray diffraction (PXRD) patterns are measured by a Siemens X-ray diffractometer (XRD, SIEMENS AG, D5000, Germany, procuring Country: Nanjing, China) (D5000). The X-ray photoelectron spectroscopy (XPS) spectrum is recorded by an Escalab250Xi photoelectron spectrometer (ThermoScientific, USA, procuring Country: Nanjing, China).

The concentration of metal ions in the degraded solution is detected by inductively coupled plasma mass spectrometry (ICP-MS, Agilent Technologies Inc., Agilent 7850 (MS), USA, procuring Country: Nanjing, China).

The surface hydrophilicity of the membrane is measured using a contact angle analyzer (DataPhysics Instruments Co., Ltd, Dataphysics DCA T21, Germany, procuring Country: Nanjing, China). The mechanical properties of the membrane are tested by an electronic automatic universal testing machine (INSTRON CORPORATION, CMT6103/ZWICK/Instron 5969, USA, procuring Country: Nanjing, China).

### 4.5. Degradability Test

At room temperature, the degradation reaction is carried out in a 50 mL centrifuge tube. A 1 cm^2^ Fe/Co-MIL-88B(NH_2_)/CaO_2_/PVDF composite membrane is immersed in 20 mL of an aqueous AFB1 or AFM1 solution (1 mg/mL) for 60 min of degradation. At specific times, 0.5 mL of the mixture is collected and filtered through a 0.22 μm polyethersulfone (PES) syringe filter, and then the AFB1 and AFM1 concentration is subsequently determined. The degradation rate (denoted as R) is calculated using the following equation:

(1) $$R(\%)=\;(C_{0}-C)\;/\;C_{0}\times 100\%$$

where *C*_0_ and *C* (mg/L) represent the AFB1 concentration in the aqueous solution at the initial reaction and the end of the reaction, respectively.

### 4.6. Metal Leaching Test

4 mg of Fe/Co-MIL-88B(NH_2_) is added to 20 mL of solution containing AFB1 (1 mg/L), while 227 μL of H_2_O_2_ (3%) solution is added for the degradation reaction. Collect 10 mL of reaction solution after membrane and Fe/Co-MIL-88B(NH_2_) degradation of toxins, centrifuge at 8000 rpm for 10 min, and analyze by ICP-MS. To demonstrate the safety of the composite membrane, the heavy metal content in the degradation solution is analyzed using ICP-MS, confirming that no nanoparticles from the composite membrane leaked into the solution.

### 4.7. Analytical Method

The sample is tested using high-performance liquid chromatography (HPLC, Shimadzu Co., Ltd., Japan) and a liquid chromatograph mass spectrometer (LC-MS, LC-MS-8050, Shimadzu Co., Ltd., Japan, procuring Country: Nanjing, China).

A Hypersil GOLD aQ C18 column (2.5 μm × 4.6 mm × 250 mm, Thermo Scientific™) is used to determine the concentration of AFB1 with HPLC (Shimadzu Co., Ltd., Japan, procuring Country: Nanjing, China). The ultra-pure water/methanol (50:50 *v*/*v*) is used as the mobile phase. The flow rate is 1 mL/min with a temperature of 35 °C. The excitation wavelength is 360 nm, and the emission wavelength is 440 nm. The total running time of HPLC analysis is 15 min, and the injection volume is 10 μL. The limit of detection (LOD) of the method is 0.1 ng/mL. The peak area is used to calculate the removal rate of AFB1.

The same C18 column is used to determine the concentration of AFM1 with HPLC using the same mobile phase, flow rate, and temperature. The excitation wavelength is 365 nm, and the emission wavelength is 435 nm. The total running time of HPLC analysis is 15 min, and the injection volume is 10 μL. The peak area is used to calculate the removal rate of AFM1.

The degradation products were determined by LC-MS. The mobile phase consists of 50% 0.1% formic acid water and 50% methanol with a flow rate of 0.3 mL/min. The chromatographic separation was carried out on a C18 column (ACQUITY UPLC BEH, 1.7 μm, 2.1 × 50 mm) with an injection volume of 10 μL. Mass spectrometry was performed in positive ion mode using an ESI source with the capillary voltage of 4.0 kV, the source temperature of 300 °C, the desolvation temperature of 526 °C, and a desolvation gas flow rate of 10 L/min. The mass spectrometer was operated in full scan mode with a scan range of *m*/*z* 100–1000 or *m*/*z* 100–500.

### 4.8. Safety Experiment

Cytotoxicological experiments are carried out by the Methyl Thiazolyl Tetrazolium (MTT) method, and HepG2 cells are selected as experimental objects to determine cell viability. HepG2 cells are seeded into 48-well plates and cultured in a carbon dioxide incubator for 24 h. Separately, the membrane samples of different areas (0.1 cm^2^, 0.5 cm^2^, 1.0 cm^2^, 1.5 cm^2^, and 2.0 cm^2^) are incubated in 200 μL of culture medium for 24 h to obtain membrane-conditioned medium. After incubation, the supernatant is collected and applied to the HepG2 cells. Subsequently, 90 μL of fresh medium and 10 μL of MTT solution (5 mg/mL) are added to the supernatant, and the mixture is incubated for 4 h. At the end, the supernatant is taken, and 100 μL of DMSO is added to each well. The plate is then placed on a low-speed shaker for 10 min to ensure that the crystals are completely dissolved. Finally, the absorbance of each well was measured at 490 nm using a microplate reader due to the instrumental equipment, as 490 nm is also commonly used in MTT assays. All measurements were performed consistently under identical conditions to ensure reliable comparisons.

The cell viability is calculated as follows:


Cell Viability (%) = (*A*_1_ − *A*_2_)/(*A*_0_ − *A*_1_) (2)

*A*_1_ represents the absorbance of the cells after incubating with different sample solutions, *A*_0_ represents the absorbance of the cells after incubating with the medium, and *A*_2_ represents the absorbance of the blank group.

To monitor residual hydrogen peroxide levels, a 1 cm^2^ Fe/Co-MIL-88B(NH_2_)/CaO_2_/PVDF composite membrane is immersed in 20 mL of a 1 mg/L AFB1 solution. Iodometric titration is used to quantify the H_2_O_2_ concentration. Every 5 to 10 min, 3 mL of the reaction solution is withdrawn, filtered through a 0.22 μm PES syringe filter to remove the composite membrane, and analyzed. The filtrate is treated with 3 mL of 0.1 M potassium hydrogen phthalate (KHP) and 3 mL of 0.4 M potassium iodide (KI), stirred, and allowed to react for 30 min. The absorbance of the resulting solution at 352 nm is measured using UV-Vis spectrophotometry.

### 4.9. Actual Test

20 mL of milk without AFB1 or AFM1 contamination is placed into a 50 mL centrifuge tube and centrifuged at 8000 rpm at −4 °C for 15 min. Subsequently, AFB1, AFM1, and a 1 cm^2^ Fe/Co-MIL-88B(NH_2_)/CaO_2_/PVDF composite membrane are added to the centrifuge tube, and the mixture is allowed to react for 60 min. Additionally, 4 mL of milk is mixed with 0.4 g of sodium chloride and 12 mL of ethyl acetate on a shaker at 300 rpm for 10 min. The mixture is then centrifuged for 10 min at 8000 rpm to separate the organic layer. The resulting 6 mL supernatant was evaporated under a nitrogen flow at 40 °C and subsequently re-dissolved in a mixture of 1 mL of 15% acetonitrile/H_2_O (V:V) and 0.1% formic acid. The concentration of AFB1 and AFM1 after degradation is determined following filtration through a 0.22 μm PES syringe filter.

To assess the potential impact of composite membrane treatment on milk quality, 15 mL of treated milk is analyzed for protein, lipid, and lactose content using standardized methods outlined by the National Food Safety Standards. Untreated milk samples serve as a control group. Three different commercially available milk brands sourced from local supermarkets in Nanjing are selected for this study.

## Data Availability

The original contributions presented in this study are included in the article/Supplementary Materials. Further inquiries can be directed to the corresponding authors.

## Supplementary Materials

The following supporting information can be downloaded at https://www.mdpi.com/article/10.3390/toxins17100516/s1: Figure S1: High-resolution XPS spectra of Fe 2p. The spectra is shown for qualitative observation; no quantitative values are implied; Figure S2: High-resolution XPS spectra of Co 2p. The spectra is shown for qualitative observation; no quantitative values are implied. Figure S3: Actual photos of different film samples. (c) PVDF, (b) PVDF+CaO_2_, (c) PVDF+Fe/Co-MIL-88B(NH_2_), and (d) PVDF+CaO_2_+Fe/Co-MIL-88B(NH_2_); Figure S4: LC-MS spectra for the AFB1 degradation; Figure S5: LC-MS spectra for the AFM1 degradation; Figure S6: Actual test of membrane; Table S1: Mechanical properties of membrane; Table S2: Comparison of different methods for AFB1 degradation; Table S3: Effect of composite membrane-treated milk samples. References [56,57,58,59,60] are cited in the Supplementary Materials.
