# Supplementary material for: Self-Sufficient Aflatoxin Decontamination System: MOF-Based Composite Membrane with Peroxidase-Mimic and Controlled H2O2 Generation
Source: Toxins (Basel). 2025 Oct 20;17(10):516. doi: 10.3390/toxins17100516 (PMC12567683; doi:10.3390/toxins17100516)
Supplement: Supplementary file 1 [file toxins-17-00516-s001.zip › toxins-3859466-supplementary.pdf]

Supplementary Material

Self-Sufficient Aflatoxin Decontamination System: MOF-Based Composite Membrane with Peroxidase-Mimic and Controlled H_2O_2 Generation

Xiaofei Cheng[a, b 1], Wenzhong Zhu[a, 1], Xueting Zhu[a, 1], Jinmin Zhang[a], Jia Yang[c], Huali Wang [d*], Xiaoqin Mo[c*], Chi Zhang[e*], Lina Wu[a*]

[a]*School of Food Science and Pharmaceutical Engineering, Nanjing Normal University, Nanjing 210023, China*

[b]*Inner Mongolia Mengniu Dairy (Group) Limited by Share Limited, Neimenggu, 010020, China*

[c]*Yangzhou Center for Food and Drug Control, Yangzhou 225002, China*

[d]*China National Center for Food Safety Risk Assessment, Beijing 10000, China*

[e]*Key Laboratory of Biotoxin Analysis & Assessment, State Administration for Market Regulation, Nanjing Institute of Product Quality Inspection, Nanjing, 210019, China*

[*] Corresponding author.

E-mail address: wanghuali@cfsa.net.cn (H. Wang); 120425254@qq.com (X. Mo); zhangchi3660@hotmail.com (C. Zhang); wuln@njnu.edu.cn (L. Wu)

[1] These authors contributed equally to this work.

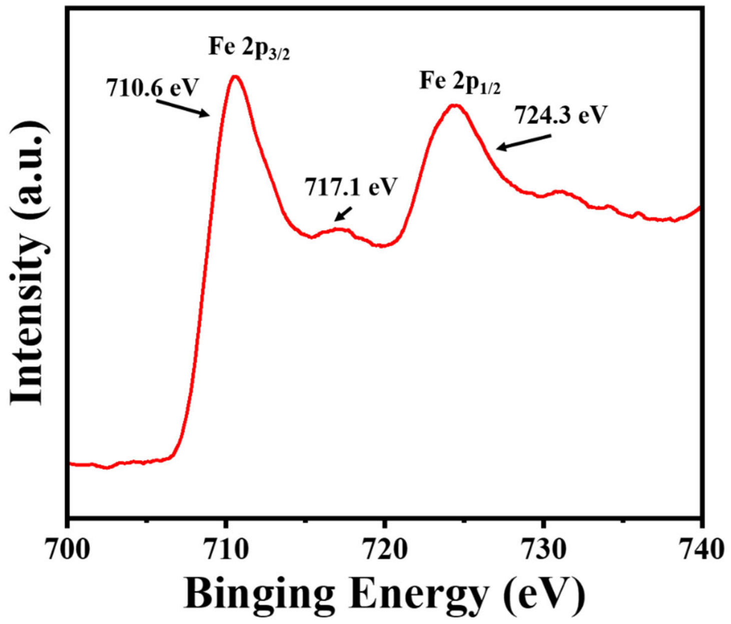

Figure S1. High-resolution XPS spectra of Fe 2p. The spectra is shown for qualitative observation; no quantitative values are implied.

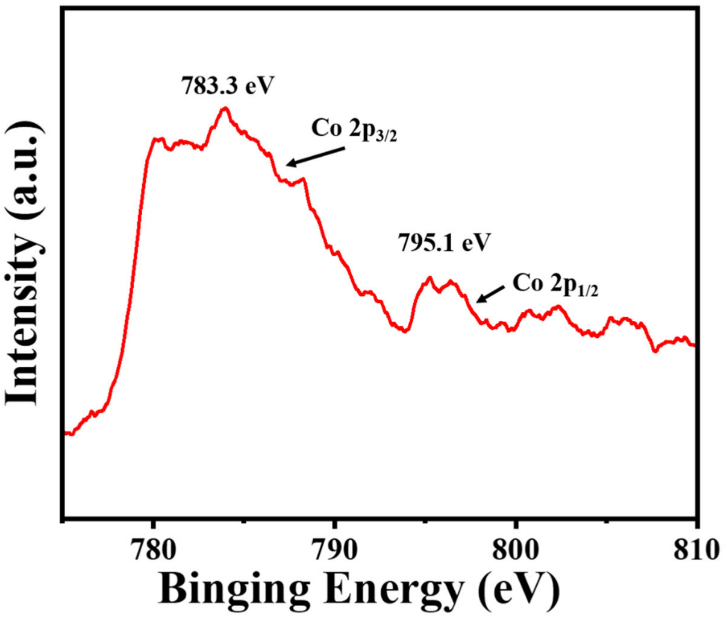

Figure S2. High-resolution XPS spectra of Co 2p. The spectra is shown for qualitative observation; no quantitative values are implied.

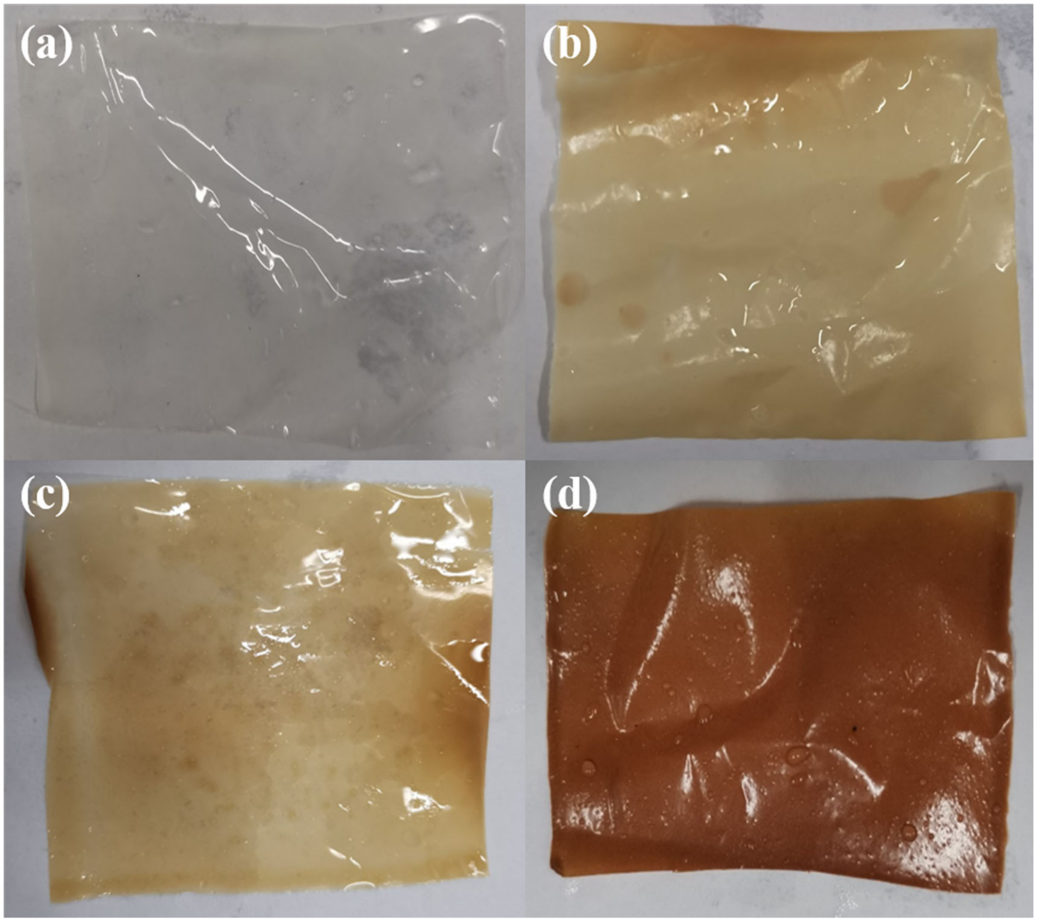

Figure S3. Actual photos of different film samples. (c) PVDF, (b) PVDF+CaO_2, (c) PVDF+Fe/Co-MIL-88B(NH_2), and (d) PVDF+CaO_2+Fe/Co-MIL-88B(NH_2).

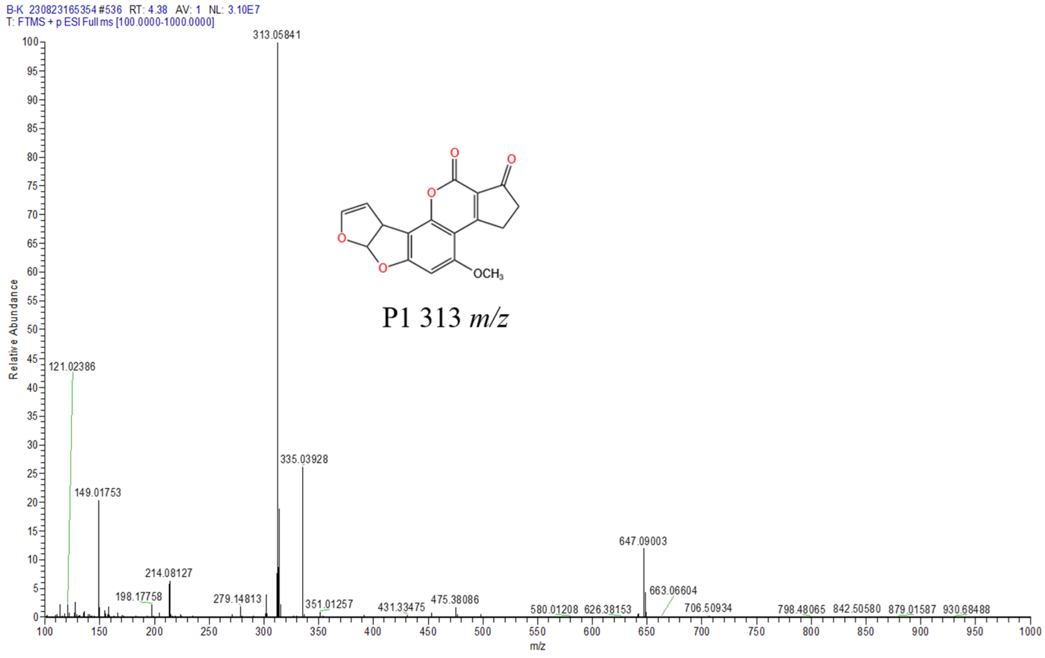

B-K 230823165354 #536 RT: 4.38 AV: 1 NL: 3.10E7
T: FTMS + p ESI Full ms [100.0000-1000.0000]
Relative Abundance
m/z
313.05841
121.02386
149.01753
335.03928
647.09003
214.08127
198.17758
279.14813
351.01257
431.33475
475.38086
580.01208
626.38153
663.06604
706.50934
798.48065
842.50580
879.01587
930.68488
OCH3
P1 313 m/z

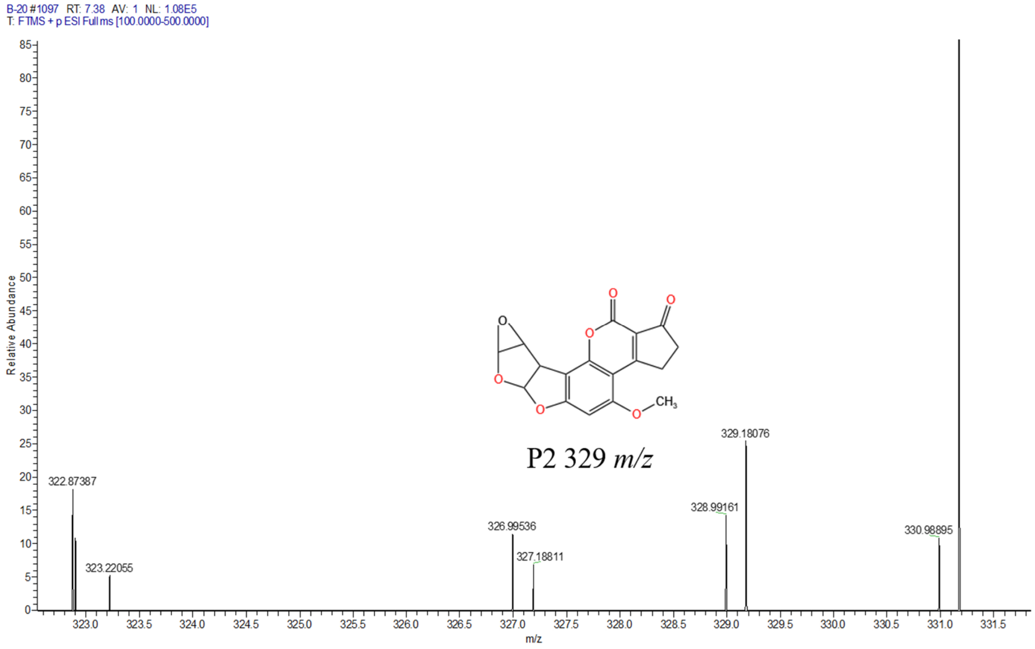

B-20 #1097 RT: 7.38 AV: 1 NL: 1.08E5
T: FTMS + p ESI Full ms [100.0000-500.0000]
Relative Abundance
m/z
322.87387
323.22055
326.99536
327.18811
328.99161
329.18076
330.98895
CH3
P2 329 m/z

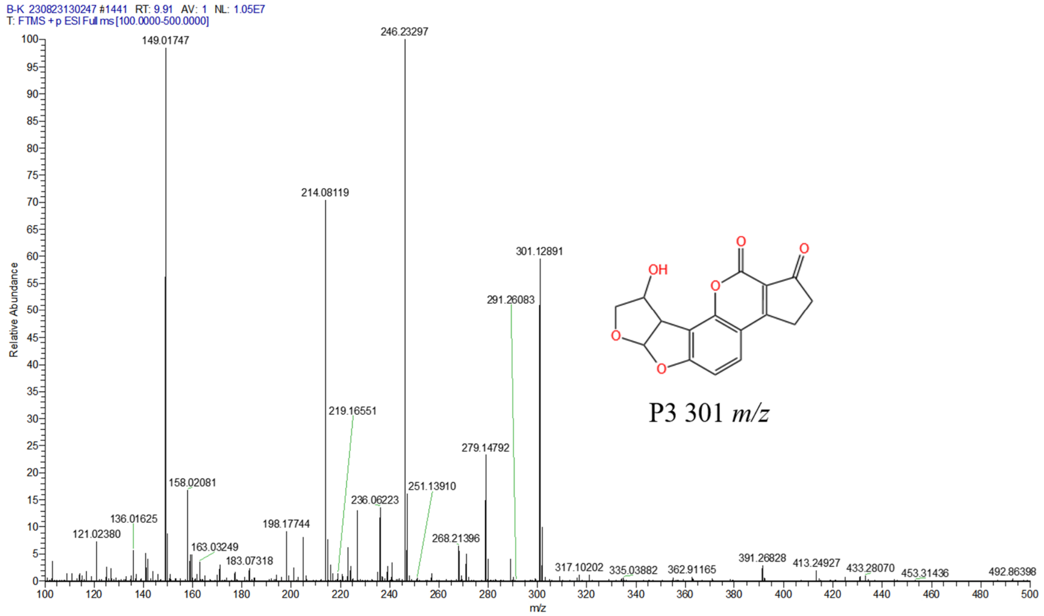
B-K_230823130247 #1441 RT: 9.91 AV: 1 NL: 1.05E7
T: FTMS + p ESI Full ms [100.0000-500.0000]
Relative Abundance
m/z
149.01747
246.23297
214.08119
301.12891
291.26083
219.16551
279.14792
158.02081
251.13910
236.06223
136.01625
121.02380
198.17744
268.21396
163.03249
183.07318
317.10202
335.03882
362.91165
391.26828
413.24927
433.28070
453.31436
492.86398
OH
O
P3 301 m/z

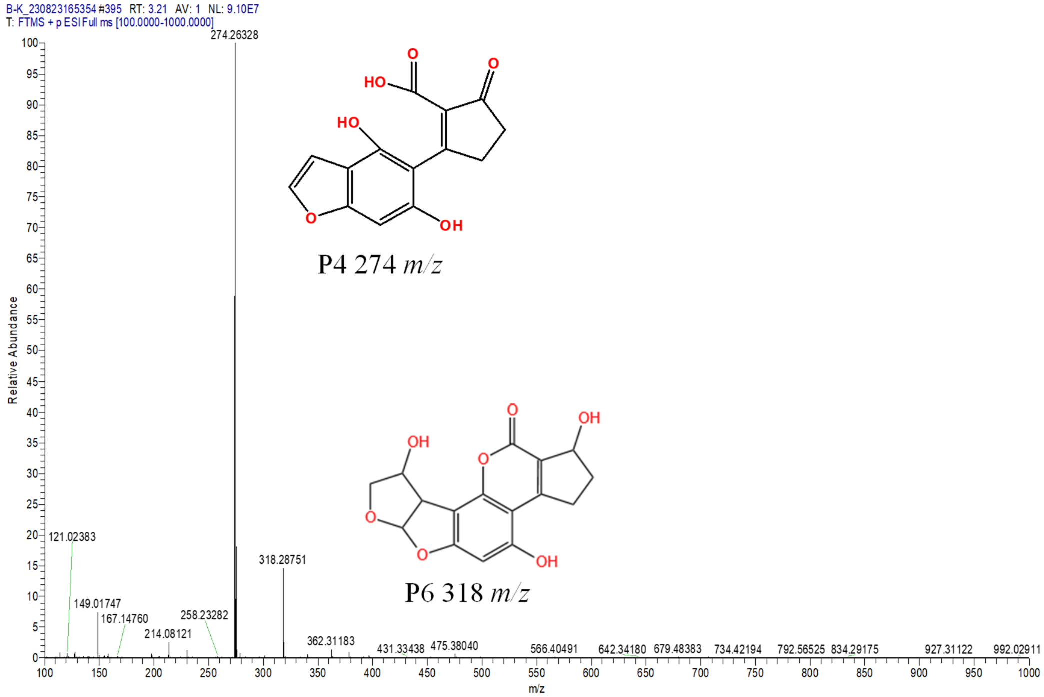
B-K_230823165354 #395 RT: 3.21 AV: 1 NL: 9.10E7
T: FTMS + p ESI Full ms [100.0000-1000.0000]
Relative Abundance
m/z
274.26328
121.02383
318.28751
149.01747
167.14760
258.23282
214.08121
362.31183
431.33438
475.38040
566.40491
642.34180
679.48383
734.42194
792.56525
834.29175
927.31122
992.02911
HO
OH
O
P4 274 m/z
P6 318 m/z

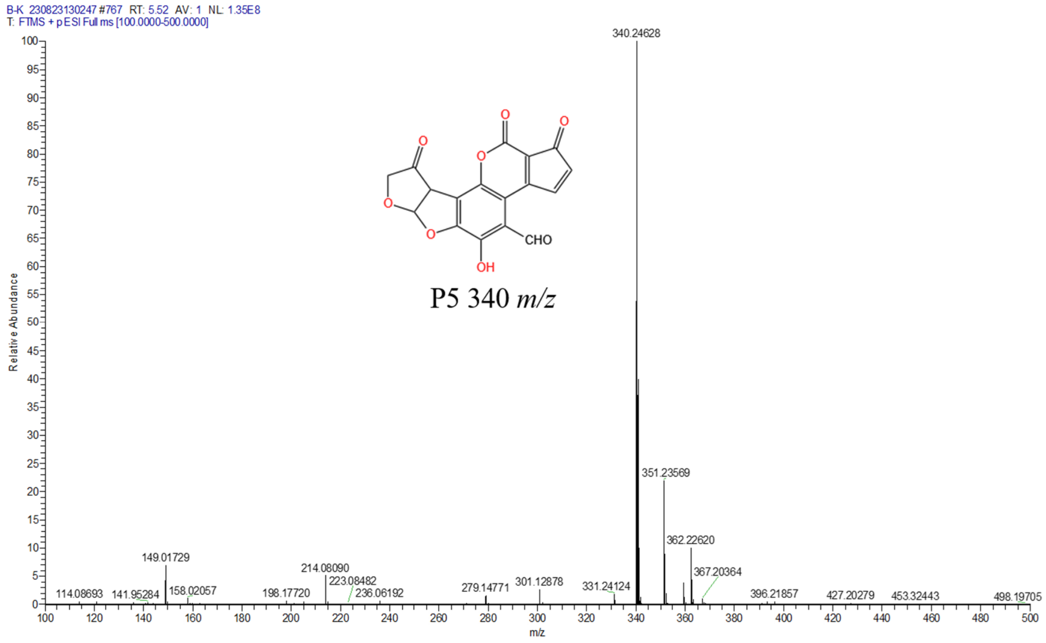

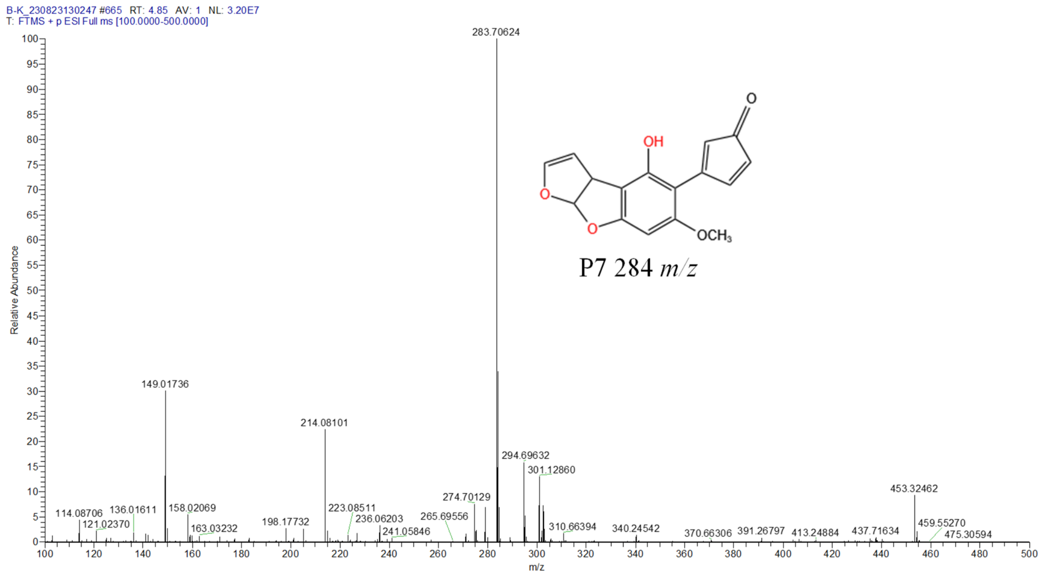

Figure S4. LC-MS spectra for the AFB1 degradation

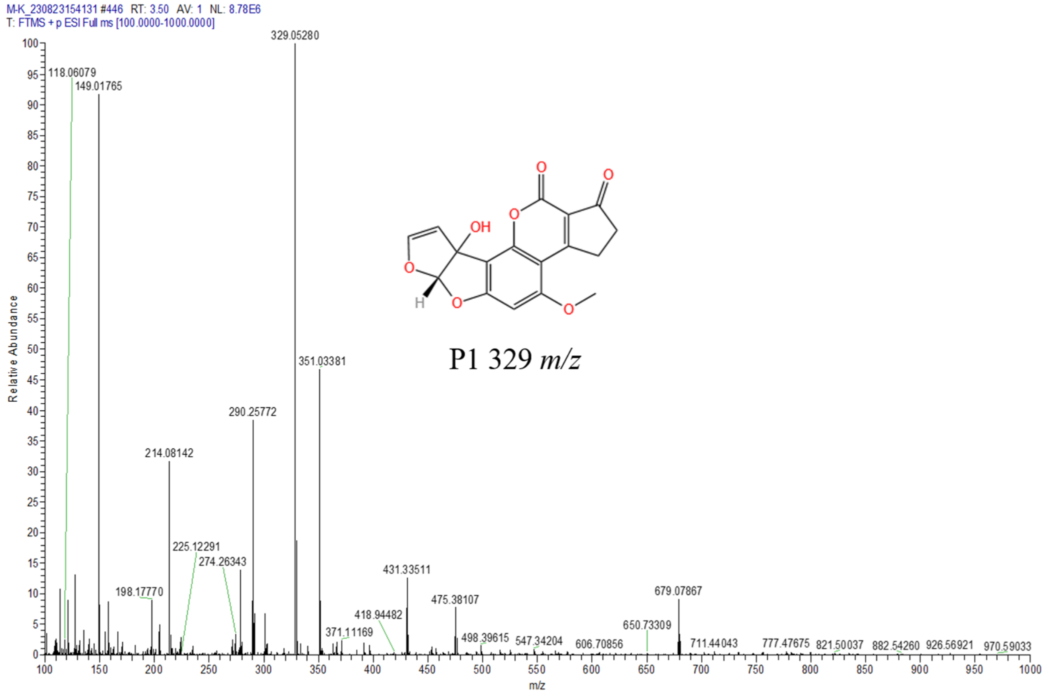
M-K_230823154131 #446 RT: 3.50 AV: 1 NL: 8.78E6
T: FTMS + p ESI Full ms [100.0000-1000.0000]
Relative Abundance
m/z
329.05280
118.06079
149.01765
351.03381
290.25772
214.08142
225.12291
274.26343
431.33511
198.17770
475.38107
679.07867
418.94482
650.73309
371.11169
498.39615
547.34204
606.70856
711.44043
777.47675
821.50037
882.54260
926.56921
970.59033
P1 329 m/z

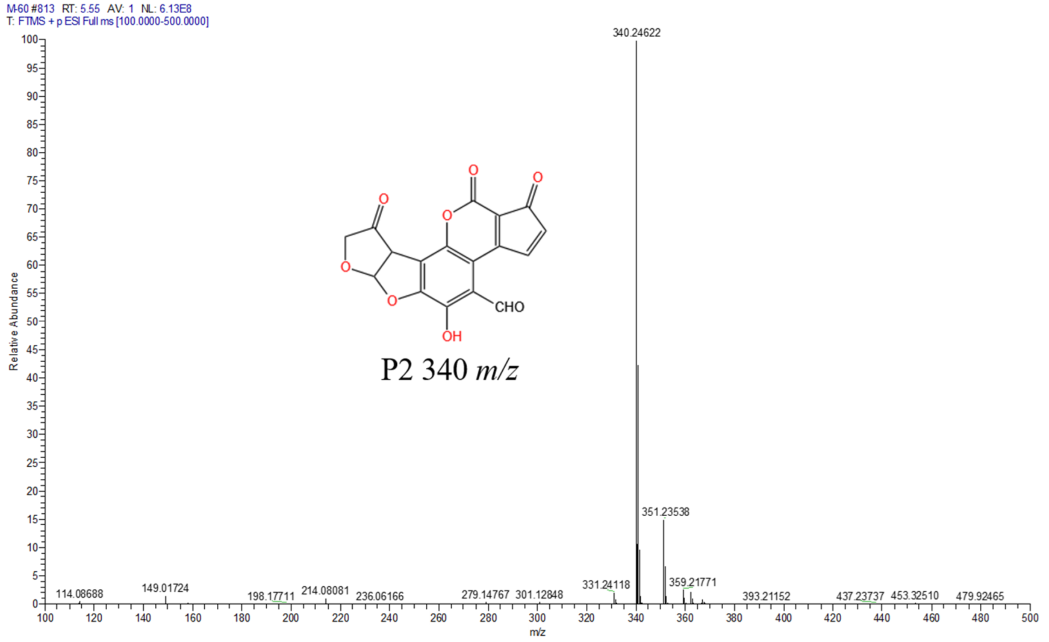
M-60 #813 RT: 5.55 AV: 1 NL: 6.13E8
T: FTMS + p ESI Full ms [100.0000-500.0000]
Relative Abundance
m/z
340.24622
351.23538
114.08688
149.01724
198.17711
214.08081
236.06166
279.14767
301.12848
331.24118
359.21771
393.21152
437.23737
453.32510
479.92465
P2 340 m/z

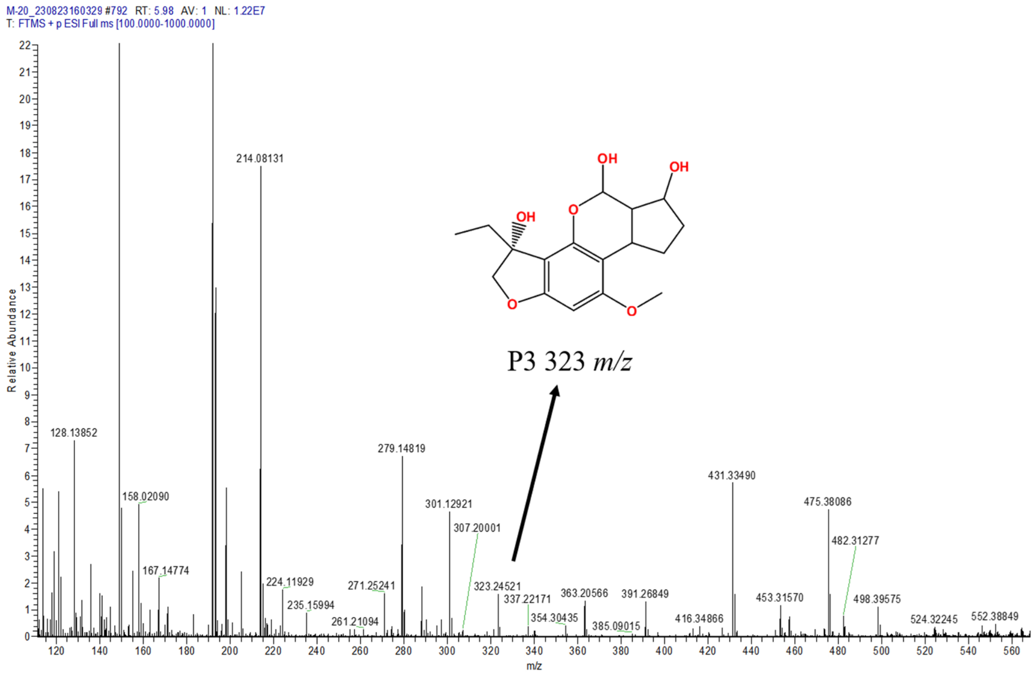

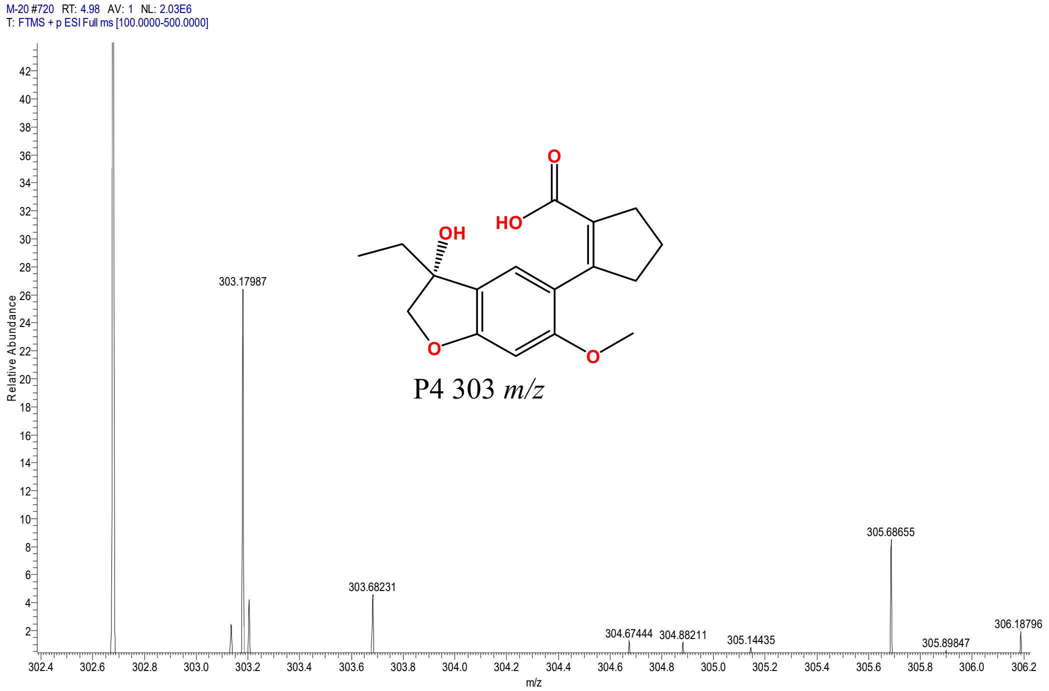

Figure S5. LC-MS spectra for the AFM1 degradation.

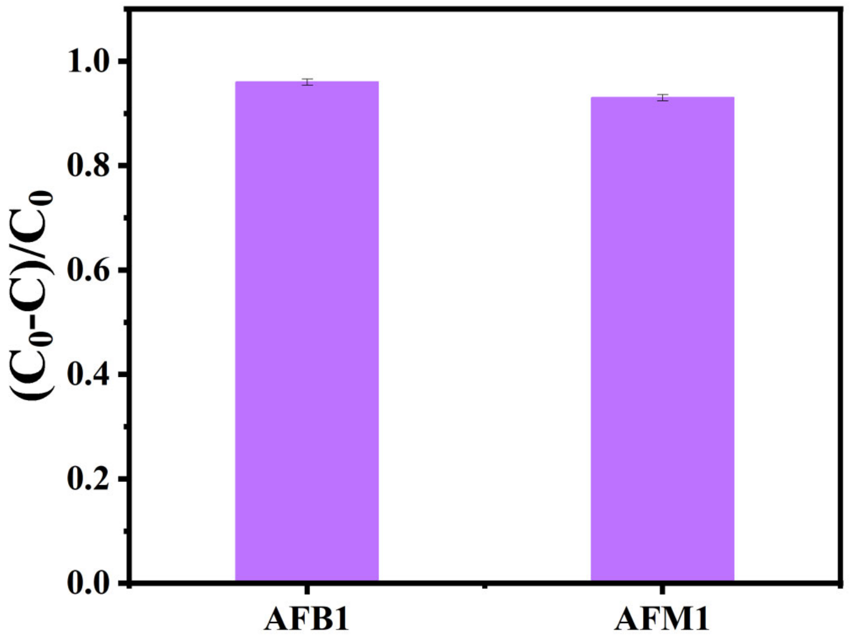

Figure S6. Actual test of membrane.

Table S1 Mechanical properties of membrane

Membrane	Elastic modulus (MPa)	Elongation at break (%)	Tensile strength (MPa)	Tensile fracture stress (MPa)	Maximum force (N)
PVDF	35.50	99.27	2.42	1.54	2.90
Ultrathin composite film	15.87	294.98	5.43	3.39	5.43

Table S2 Comparison of different methods for AFB1 degradation.

Methods	Toxin	Degradation rate (%)	Time (h)	Reference
Photochemical	AFB1 AFM1	65% 78%	-	[56]
MIPs	AFM1	87.3%-96.2%	-	[57]
Fermentation	AFB1	82.8%	72 h	[58]
Enzyme	AFB1	85.61%	24 h	[59]
Enzyme	AFB1	81.14%	2 h	[60]
Enzyme	AFB1 AFM1	＞95%	1 h	This work

Note: References are included in the main text.

Table S3 Effect of composite membrane-treated milk samples.

Milk quality	milk sample	membrane-treated milk sample
Protein (g/100 mL)	3.53	3.54
Lipid (g/100 mL)	3.19	3.17
lactose content (g/100 mL)	4.62	4.59
